# Disturbed Sleep in PTSD: Thinking Beyond Nightmares

**DOI:** 10.3389/fpsyt.2021.767760

**Published:** 2021-11-24

**Authors:** Marike Lancel, Hein J. F. van Marle, Maaike M. Van Veen, Annette M. van Schagen

**Affiliations:** ^1^Centre of Expertise on Sleep and Psychiatry, GGZ Drenthe Mental Health Institute, Assen, Netherlands; ^2^Department of Clinical Psychology and Experimental Psychopathology, University of Groningen, Groningen, Netherlands; ^3^Department of Psychiatry, Amsterdam Neuroscience, Vrije Universiteit, Amsterdam UMC, Amsterdam, Netherlands; ^4^GGZ InGeest Specialized Mental Health Care, Amsterdam, Netherlands; ^5^ARQ Centrum'45, Oegstgeest, Netherlands

**Keywords:** PTSD, sleep, sleep disorders, nightmares, insomnia, sleep apnea, assessment, treatment

## Abstract

Sleep disturbances frequently co-occur with posttraumatic stress disorder (PTSD). Insomnia and nightmares are viewed as core symptoms of PTSD. Yet, relations between disturbed sleep and PTSD are far more complex: PTSD is linked to a broad range of sleep disorders and disturbed sleep markedly affects PTSD-outcome. This article provides a concise overview of the literature on prevalent comorbid sleep disorders, their reciprocal relation with PTSD and possible underlying neurophysiological mechanisms. Furthermore, diagnostic procedures, standard interventions—particularly first choice non-pharmacological therapies—and practical problems that often arise in the assessment and treatment of sleep disturbances in PTSD are described. Finally, we will present some perspectives on future multidisciplinary clinical and experimental research to develop new, more effective sleep therapies to improve both sleep and PTSD.

## Introduction

Sleep disturbances frequently occur in posttraumatic stress disorder (PTSD) and are reported by 70–90% of patients (1). Nightmares (intrusions) and difficulties sleeping (hyperarousal) are specifically included in the diagnostic (DSM-5) criteria of the disorder (2). In addition, various other sleep disorders are common in PTSD [e.g., (3, 4)]. It has long been thought that interventions focusing on trauma itself would eventually reduce disturbed sleep, but accumulating evidence shows that sleep disorders play a central role in both the development and maintenance of PTSD [e.g., (5, 6)] and therefore require particular clinical attention.

In this paper we provide an overview of prevalent sleep disorders in PTSD, the reciprocal association of sleep disturbances and PTSD and its underlying mechanisms, as well as information on accurate assessment and treatment of disturbed sleep tailored to the PTSD patient population. Finally, our perspectives for future research directed at more effective sleep-targeted interventions and integrated treatment strategies are described. Our aim is to enhance awareness of clinical practitioners of the importance of targeting sleep in PTSD treatment.

## PTSD and Sleep Disturbances

The majority of patients with PTSD, about 50–70%, suffer from recurrent distressing nightmares (see Table 1 for an overview of the most frequently occurring sleep disorders in PTSD, their characteristics, ways of assessment and treatment). These can be exact replications or more symbolic representations of traumatic experiences, and primarily occur during rapid eye movement sleep (REMS) (7). Insomnia symptoms, reported by ~70% of patients (8), are often related to increased autonomic arousal and fear of sleep: fear of loss of control and/or of having nightmares (9, 10). PTSD is also associated with obstructive sleep apnea (OSA), concerning 40–90% of PTSD patients (11). The repeated OSA events lead to frequent oxygen desaturations and arousals. Insomnia, nightmares and OSA may trigger and exacerbate each other, forming a vicious cycle (1, 4). In addition, multiple studies found a high proportion (around 33%) of periodic limb movement disorder (PLMD) in PTSD patients (12). The limb movements during sleep are associated with arousals/awakenings. Also relatively prevalent in PTSD are periods of sleep paralysis, typically occurring during (REM) sleep-wake transitions, which are often accompanied by distressing experiences, referred to as hypnagogic or hypnopompic hallucinations (13). Although the exact frequency is unclear, PTSD is also linked to remarkable disruptive nocturnal behaviors, including abnormal vocalizations and complex body movements. These parasomnias are generally thought to occur during non-REMS (confusional arousals, night terrors, sleepwalking), but may also take place during REMS, implying REMS behavior disorder (RBD)-like periods of REMS without the usual muscle atonia (RWA) with dream enactment. Mysliwiec et al. (14) proposed a distinct trauma-associated sleep disorder (TASD), conceptualized as a parasomnia encompassing nightmares, disruptive nocturnal behaviors as well as RWA [see (15) for an illustrative case study]. In support of this idea, a recent study in a large sample of veterans found self-reported dream enactment in nearly 40%. However polysomnography (PSG) showed no RWA in 80% of this group, indicating a non-REMS parasomnia, rather than a REMS phenomenon (16). Furthermore, in those veterans with RWA, RBD appeared related to PTSD (prevalence rate 15%) and even more so to the combination of PTSD and traumatic brain injury (prevalence rate 21%). Therefore, it is still controversial whether TASD really represents a separate sleep disorder (17, 18).

**Table 1:** Overview of frequently occurring sleep disorders in PTSD: characteristics, assessment and treatments.

**Sleep disorder**	**Timing and sleep-phase**	**Duration**	**Behavior during**	**Behavior after**	**Recollection of the event**	**Provocative factors**	**Assessment**	**PSG changes**	**Treatment**
**Non-REMS**									
Confusional arousals (CA)	First third of the night, first bout of slow wave sleep (SWS)	Seconds to minutes	Sudden arousal, followed by confusion, disorientation, eyes open	Confusion	Amnesia	Sleep deprivation, fever, anxiety, stress, sleep apnea, sleep-related movement disorder, caffeine	Observations by bed partner, video observation, PSG optional	Arousal out of SWS, return to sleep	Avoidance of provocative factors, sleep hygiene
Sleepwalking (somnambulism)	See CA	1–10 mins	Abrupt arousal, motor activity outside the bed, possibility of confusion/agitation when suddenly interrupted	Sleeping again	Amnesia	See CA. Hypnotic zolpidem	See CA	See CA	See CA. Safety measures for protection: remove sharp objects, lock windows and doors. If dangerous, consider pharmacotherapy
Night terrors (pavor nocturnus)	See CA	Seconds to minutes	Sudden arousal with intense screaming, inconsolable crying or agitation, and increased autonomic discharge	If awake: being anxious	Amnesia	See CA	See CA	Sudden and incomplete arousal from SWS	See CA. Also psycho-education to parents/partners and patients that episodes are transient and patient should not be awakened. Installation of fixed wake-up times prior to episode, stress reduction training.
Periodic limb movements (PLMs)	Non-REMS	Seconds	Repetitive cramping or jerking of the legs during sleep	Continue sleeping, possible short arousal	Amnesia / no recollection	Somatic disease (including iron deficiency), smoking, caffeine, medication use, sleep apnea	Observations by bed partner, video observation, PSG	Consecutive bursts of activity in leg muscles, with or without arousals	Sleep hygiene, avoidance of possible triggers, when severe with frequent arousals: pharmacological treatment
**REMS**									
Sleep paralysis	Transition from REMS to wakefulness	<1 min	Enduring muscle atonia: not being able to talk and move body and limbs when waking up (less frequently when falling asleep), anxiety	Sometimes anxious reaction, paranormal sensations	Recollection	Sleep deprivation, schedule disruption, alcohol ingestion	Self-report, possible PSG	Persistence of consciousness and alpha activity intruding into the otherwise desynchronized REMS EEG	Psycho-education and reassurance. Paralysis usually resolves in <1 min and/or after sensory stimulation (touch). Focus on small movements, such as breathing and eye movement
Nightmares/nightmare disorder	During REMS, last third of the night	Seconds to minutes	Vivid and extended extremely dysphoric dreams, with a strong negative emotional tone, typically involving threats to security, physical and/or emotional integrity; muscle atonia	Sudden and violent awakening, often accompanied by anxiety, sometimes shortness of breath. Fear of going back to sleep	Clear recollection of dream content and storyline	Sleep deprivation, fever, stress, major (traumatic) events, medications such as antihypertensives, antidepressants, and dopamine agonists	Self-report, nightmare logs	Densely packed eye movements during REMS	Sleep hygiene, stress reduction, imagery rehearsal therapy (IRT): rescripting of nightmares, imaginal exposure to nightmare content, consider pharmacotherapy: prazosin
REMS behavior disorder (RBD)	During REMS, last third of the night	Seconds to minutes	Loss of REMS atonia. Dream enactment motor activity: usually trying to prevent an attack, and any behavior that could occur during a dream, possibility of injuring themselves and/or bed partner	Awakening often accompanied by anxiety, sometimes shortness of breath. Fear of going back to sleep, fear of hurting bed partner	Vivid recollection of the dream, correlating with observed behavior	Acute phase: medication induced: tricyclic antidepressants, monoamine oxidase inhibitors, and serotonin reuptake inhibitors; alcohol withdrawal, benzodiazepine withdrawal	Self-report, observations by bed partner, PSG	REMS without atonia (RWA)	Bedroom safety principles, removing provocative factors, consider pharmacotherapy: melatonin, clonazepam
**Other**									
Sleep-related hallucinations	When falling asleep (hypnagogic) or waking up (hypnopompic)	Seconds to 1 minute	Hallucinations with visual, auditory, tactile, olfactory, and/or kinetic properties, possible paranormal sensations, sometimes in combination with sleep paralysis	Sometimes fear, paranormal beliefs	Recollection	Sleep deprivation, daytime naps, psychoactive substances: opiates, cannabis, amphetamines, cocaine, hypnotics, and zopiclone	Self-report	Not known	Psycho-education
Sleep talking (somniloquy)	Mostly in non-REMS, also in REMS	Seconds to minutes	Talking in own language or nonsense, one word or an extensive dialogue	Sleep continues	Amnesia	Anxiety, sleep deprivation and fever	See CA	Occurring in both non-REMS and REMS	Psycho-education, sleep hygiene and stress reduction
Insomnia	Entire night	1–8 h	Lying awake, unrest, rumination	Daytime fatigue, concentration problems, impaired emotion and trauma regulation	Recollection	Arousal, negative thoughts, fear of nightmares, trauma-related triggers, such as bed, bedroom, nighttime, darkness	Self-report, sleep diary, possible PSG	Longer periods of wakefulness, frequent awakenings. REMS fragmentation with very short arousals	CBT-I
Obstructive sleep apnea syndrome (OSAS)	Entire night	1–8 h	Short breathing stops, and arousals without conscious awakening	Continue sleeping, daytime fatigue, non-refreshing sleep, possible development of insomnia	Amnesia	Obesity, snoring, smoking, use of alcohol or other sedating substances/ medication	Observations by bed partner, audio/video recording, PSG	Recurrent partial or complete cessation of air flow, with hypoxia and arousals/sleep fragmentation	Weight loss, sleep hygiene, avoidance of possible triggers, CPAP, MRA, position trainer, ENT surgery
Restless legs syndrome (RLS)	Prior to sleep	Minutes–hours	Uncomfortable sensations in legs (sometimes arms) while awake; irresistible urge to move limbs	Awake	Recollection	Somatic disease (including Iron deficiency), smoking, caffeine, medication use	Self-report	Longer sleep onset latency, often co-occurring PLMs during sleep	Sleep hygiene, avoidance of possible triggers, pharmacological treatment

## Interrelations Between PTSD and Sleep Disturbances

Research strongly indicates that disturbed sleep is not merely a symptom or consequence of PTSD, but constitutes a predisposing, precipitating and perpetuating factor for PTSD. Sleep disturbances prior to and/or shortly after trauma increase the risk for PTSD (5, 12). For instance, in patients admitted to an emergency department after a motor vehicle collision both pre-trauma insomnia and nightmares predicted subsequent PTSD development (19). Furthermore, sleep disturbances affect the clinical course of PTSD: poor sleep quality is associated with reduced responsiveness to trauma-focused therapy [e.g., (20, 21)], while interventions targeting insomnia, nightmares or OSA improve sleep quality and ameliorate daytime PTSD symptoms (22). For example, Kanady et al. (9) observed that cognitive behavioral treatment of insomnia (CBT-I) in patients with PTSD and insomnia significantly decreased hypervigilance as well as PTSD symptom severity, and both were related to persistent reductions in fear of sleep. Moreover, sleep disturbances often persist after trauma-focused therapy (10). For example, Walters et al. (23) recently showed that prolonged exposure therapy improved daytime PTSD symptoms, but did not ameliorate insomnia and nightmares in veterans with PTSD. Residual insomnia has been shown to be an important risk factor for the development of and relapse in diverse mental disorders [e.g., (24)].

The reciprocal relations between sleep disturbances and PTSD suggest that disturbed sleep constitutes a causal factor in PTSD (25, 26). This causality is partly based on sleep's role in memory consolidation and emotion regulation (27, 28). While memory consolidation takes place during both slow wave sleep (SWS; deep non-REMS) and REMS, the processing of emotional memories is thought to happen primarily during REMS (29). In PTSD, traumatic memories arise in part from a failure in extinction learning, i.e., learning that the previously conditioned stimulus no longer represents a threat (30, 31). It is postulated that REMS disturbances, resulting from the noradrenergic hyperactivation typical of PTSD, hamper the consolidation of extinction memory, leading to a failure of the extinction memory to persist and generalize (32). So far the experimental support for this idea is limited, but nonetheless growing. Sleep disturbances following a traumatic event, including fragmented REMS, predict the development of PTSD (33–35). In polysomnographic studies, PTSD is characterized by reduced SWS and increased REM density (36, 37) as well as REMS fragmentation (38). These characteristics may well result from increased noradrenergic tone during (REM) sleep in PTSD patients (39, 40). Focusing on the role of sleep in the treatment of PTSD, a recent study found that the level of SWS and REM density positively predict treatment outcome (41). This and other clinical studies point toward an additional role of non-REMS disturbances, particularly a shortage of SWS, in the development and perpetuation of PTSD. Furthermore, shared neuromodulatory pathways may also underlie the relationship between PTSD and disturbed sleep. Especially (hyperactive) noradrenergic projections from the locus coeruleus (LC), as part of both the sleep-wake and PTSD-related circuitry, could form a final common pathway in generating the state of hyperarousal typical for both PTSD and disturbed sleep (32). Insomnia (42), nightmares (26) and most other sleep disorders discussed in this perspective are characterized by hyperarousal, frequent disruptions in REMS and aberrant LC-firing. In case of OSA, trauma-related hyperarousal may promote sleep disordered breathing (43). Vice versa, untreated OSA may contribute to development of PTSD, being a continuous stressor leading to sympathetic overactivity and disruption of sleep (44). As OSA events often occur during REMS, it is the brain's capacity to process negative emotions during REMS that is most likely affected.

## Assessment of Sleep Disturbances in PTSD

Sleep disturbances can be screened and assessed with a clinical interview and objectified with other measures such as actigraphy and PSG. An actigraph and/or smartwatch can be helpful in detecting nightly arousals and limb movements, as well as daily rhythms in sleep and activity, and estimating sleep onset latency, total sleep time and sleep efficiency (45). PSG (with/without overnight video recording) provides an accurate picture of multiple physiological parameters related to sleep and wakefulness. PSG is less suitable as a screening tool, because it is an elaborate measurement which might not be readily accessible and financially feasible.

For an accurate diagnosis of PTSD according to DSM-5 criteria (2), the Clinician Administered PTSD Scale (CAPS-5) (46) can be used. It is a structured interview to diagnose current and life-time PTSD. However, the CAPS-5 is not sufficient for assessing the presence of sleep disorders, as it contains only two questions regarding sleep problems, considering nightmares and sleep disturbance in general. Diagnoses of sleep disorders are easily missed if specific diagnostic criteria are not inquired about. Therefore an accurate clinical assessment according to the International Classification of Sleep Disorders 3 (ICSD-3) (47) of sleep history, present sleep quality, sleep-wake behavior (preferably including information from the bedpartner to get a more accurate report of nightly behaviors) and screening for sleep disorders is essential.

We recommend an extensive clinical interview as there is no comprehensive questionnaire for screening diverse sleep disturbances in PTSD available. The diagnostic procedure should include an assessment of daily routines, diet, substance (ab)use, medication, mental state, presence of diseases and/or pain (or other physical limitations that compromise sleep), activity levels during night and day, and sleep behaviors including fear of sleep (10) [see (48) for a comprehensive review of the assessment and treatment guidelines of insomnia].

In PTSD the following events should be evaluated. (1) Presence of trauma-related triggers associated with sleep, the bedroom, nighttime and/or darkness, as these triggers might maintain a high arousal level, thereby hampering sleep onset and sleep maintenance. (2) Evaluation of circadian rhythm sleep-wake disorders in (uniformed) personnel working irregular hours (military personnel, police officers, fire-fighters, first responders). (3) Presence of parasomnias and distinguishing the different parasomnias, which is important for psychoeducation as well as treatment indication. For the detection of nightmares, which occur primarily during REMS, screening questionnaires such as the Nightmare Disorder Index (NDI) might be useful (49). However, both patients with PTSD and clinicians tend to misinterpret all nightly behaviors/experiences as nightmares. As the NDI does not cover other parasomnias, the clinician should always ask further about the experiences. Non-REMS parasomnias, such as confusional arousals, night terrors and sleepwalking, are often misdiagnosed as nightmares. Experiences during non-REMS parasomnias are generally not remembered well. The associated emotional distress can therefore be different from nightmares that are typically remembered vividly. It is important to ask patients to describe their nightmares in detail: What is the story in the dream? Is this trauma-related or more symbolic? What is the emotional intensity? Other parasomnias, such as sleep paralysis with or without hypnagogic and/or hypnopompic hallucinations, can be distressing, but they are not the same as nightmares. (4) Patient and bedpartner need to be asked about snoring, breathing stops, arousals and other symptoms to screen for OSA. One should take into account that the usually reported excessive daytime sleepiness is often not experienced by PTSD patients with OSA, possibly due to hyperarousal, yielding low scores on a screening questionnaire such as the Epworth Sleepiness Scale (50). An overnight audio-recording can be a useful tool to screen for sleep-related breathing problems. However, a PSG is the most objective measurement to assess OSA and its severity (51). (5) Patients and bedpartner can be asked about movements during sleep, and if present these movements can be objectified and interpreted with video-assisted PSG.

## NON-Pharmacological Treatment of Sleep Disturbances in PTSD

With or without PTSD, non-pharmacological interventions are first choice in the treatment of insomnia, nightmares and other (non-REMS) parasomnias (48, 52–54). In line with this, a recent meta-analysis on studies in PTSD patients found that PTSD symptoms and sleep both improve across all PTSD and sleep treatments. Yet, sleep improved the most after sleep-focused interventions, especially psychotherapy approaches (55).

### Insomnia

For insomnia CBT-I has shown the most evidence of efficacy (56). CBT-I consists of several therapeutic components targeting different aspects of the sleep disorder: psychoeducation about sleep and sleep hygiene, relaxation training, behavioral interventions such as stimulus control (focus on re-connection of bed/bedroom with sleep) and sleep restriction (focus on reduction of time in bed to total sleep time), and cognitive therapy (48). Drawn from clinical experience and the cognitive behavioral model of PTSD, the following interventions within CBT-I require specific attention in PTSD: relaxation training because of hyperarousal (57); treatment of trauma-related triggers associated with sleep, the bed and/or bedroom, with exposure *in vivo*, EMDR and/or cognitive therapy. Furthermore, other interventions promoting the feeling of safety, such as a photograph of a loved one next to the bed, sleeping with a dim light, soothing music or white noise can be helpful. An increasing number of studies in patients with both PTSD and insomnia show positive effects of CBT-I on sleep efficiency, time awake after sleep onset, self-reported insomnia severity and fear of sleep (58). Another practice based intervention is the use of weighted blankets, some patients benefit from it. It is a simple non-invasive intervention and a first trial shows promising results (59). However, the presence of OSA is a contra-indication.

### Nightmares

If nightmares are particularly prominent and perpetuate fear of sleep and insomnia, one can decide to treat nightmares before starting trauma-focused therapy. Most evidence is found for imagery rehearsal therapy, a technique for rescripting the nightmare story toward a better ending (60). The new dream is subsequently rehearsed through imagination. Imaginal exposure to the nightmare story is another effective, however, less studied intervention (53). There are no studies on EMDR for nightmares, even though it can be argued that desensitization of the nightmare image might be helpful.

### Night Terrors or Arousals

If the patient has night terror-induced arousals, the bedpartner can sooth the patient with a soft and low voice, directing him/her back to bed and to sleep. Do not force awakening, ensure safety and trust that the patient will have no recollection of the event. If the arousals occur often and generally at the same time of the night it can be helpful to awaken the patient 15–30 mins before the expected arousal to prevent its occurrence (61).

### Obstructive Sleep Apnea

Continuous positive airway pressure (CPAP) and mandibular repositioning appliance (MRA) can be used, and show most evidence in the treatment of OSA syndrome. CPAP has been shown to successfully reduce PTSD symptoms, including nightmare frequency, possibly by stabilizing the arousal system (43). In veterans with subclinical PTSD, non-compliance to CPAP therapy leads to increased PTSD symptoms, implying that optimal OSA-treatment prevents progression to clinical PTSD (44). If OSA-treatment adherence, e.g., wearing a CPAP-mask or MRA, is complicated by trauma-related anxiety, this needs to be specifically addressed, for example with cognitive therapy or EMDR. Other treatment options may be considered, such as weight reduction.

### Timing of Sleep Interventions

There is no guideline available for the timing of sleep-targeted interventions in PTSD in relation to trauma-focused therapy. Because of the reciprocal relation between PTSD and sleep disturbances one can argue that the sequence of interventions should be determined by the most prominent symptoms. Moreover, regarding the heterogeneity of PTSD symptoms, it is unlikely that a “one size fits all” treatment will be found. Therefore, we recommend focusing the treatment on the most distressing symptoms and/or administer two different treatments, e.g., EMDR for PTSD and CBT-I for sleep disturbances, side by side. Through monitoring the treatment process, the treatment plan can be adjusted when necessary.

## Pharmacological Treatment of Sleep Disturbances in PTSD

Several types of drugs have been specifically evaluated in PTSD-related sleep disorders (51). Alpha1-receptor antagonists such as prazosin are best supported by evidence, showing improvement in nightmares as well as insomnia (62, 63). Both sedating antipsychotics and antidepressants have been found beneficial in the treatment of PTSD, including specific positive effects on sleep quality and nightmares, but need close monitoring of negative effects such as hang-over, metabolic dysregulation, and induction/elevation of restless legs syndrome (RLS), PLMD and nightmares (64). The use of benzodiazepine-receptor agonists is controversial in patients with PTSD, not just because of generally known adverse effects, but specific negative outcomes such as worse therapy outcomes and increased risk of developing PTSD when used directly following trauma (65). Considering current evidence, pharmacological treatment of insomnia and nightmares in PTSD should be regarded as temporary and additional, rather than alternative, to psychological interventions.

## Conclusions and Perspectives

Research convincingly demonstrates that PTSD is frequently associated with multiple and diverse sleep disorders that impact both PTSD development, maintenance and recovery. Thus, an early and comprehensive assessment of comorbid sleep disorders as well as their timely treatment is of high clinical relevance for patients with trauma and PTSD. In our opinion, centers providing (mental) health care to patients with PTSD should, therefore, include at least one clinician trained in sleep medicine and establish close collaboration with a sleep center for accurate assessment and (interdisciplinary) treatment of co-occurring sleep disorders.

Yet, there are clear gaps in the knowledge on the links between PTSD and sleep and to optimize PTSD-outcome further research and innovations are warranted. For both research and clinical practice, it would be helpful to develop a screening instrument to more accurately assess all sleep disturbances and contributing factors relevant in PTSD populations, ultimately leading to a guideline for the assessment of sleep disorders in PTSD. Prospective studies of large, naturalistic cohorts suffering from trauma implementing both subjective and objective sleep measures, would be highly informative for instance with respect to delineating the sleep-related protective as well as risk factors in the development of PTSD. Furthermore, evidence on the efficacy of integrated PTSD and sleep treatment is limited to small samples, specific patient groups (veterans) and only a few sleep disorders (insomnia and nightmares) and interventions. Research needs to be expanded to include larger and more diverse groups of traumatized/PTSD patients (to entangle general and population-specific factors) and diverse, both pharmacological and non-pharmacological, treatment strategies for all relevant sleep disorders. Moreover, novel developments in the neuroscience of sleep may also guide PTSD treatment. Combining for instance trauma-focused treatment with new EEG-based techniques to deepen and lengthen SWS (66, 67) could have a synergistic effect through enhanced consolidation of the traumatic memories altered in therapy. Due to faster and more complex oscillatory dynamics, such sleep-based interventions are harder to perform during REMS. Alternatively, novel behavioral methods to strengthen memories during sleep (known as targeted memory reactivation, TMR) (68, 69) could in theory be used in PTSD during post-treatment sleep to augment treatment outcome (70).

## Data Availability Statement

The original contributions presented in the study are included in the article/supplementary material, further inquiries can be directed to the corresponding author.

## Author Contributions

All authors listed have made a substantial, direct, and intellectual contribution to the work and approved it for publication.

## Conflict of Interest

The authors declare that the research was conducted in the absence of any commercial or financial relationships that could be construed as a potential conflict of interest.

## Publisher's Note

All claims expressed in this article are solely those of the authors and do not necessarily represent those of their affiliated organizations, or those of the publisher, the editors and the reviewers. Any product that may be evaluated in this article, or claim that may be made by its manufacturer, is not guaranteed or endorsed by the publisher.
